# Profunda femoris vein laceration caused by a displaced lesser trochanter fragment: a case report of intraoperative vascular risk in intertrochanteric fracture

**DOI:** 10.3389/fsurg.2026.1776045

**Published:** 2026-04-10

**Authors:** Mei-Ren Zhang, Xiao Zeng, Kui Zhao, Jian-Hao Guan, Hai-Yun Chen

**Affiliations:** 1 Orthopedics Trauma Department, ZhuHai Hospital, Guangdong Provincial Hospital of Chinese Medicine, Zhuhai, Guangdong, China; 2 Guangzhou University of Chinese Medicine Second Clinical College, Guangzhou, China

**Keywords:** displaced lesser trochanter spike, femoral vein injury, intertrochanteric femoral fractures, intraoperative vascular risk, laceration

## Abstract

**Background:**

Vascular injury is a rarely reported complication of intertrochanteric fractures, with most literature focusing on arterial involvement. This case report describes an even rarer occurrence: a profunda femoris vein laceration caused by a displaced lesser trochanteric fragment, highlighting a potentially underrecognized intraoperative risk.

**Case presentation:**

We describe an 83-year-old male with an intertrochanteric fracture (AO/OTA 31-A2.2) in whom a displaced lesser trochanteric fragment was found in direct contact with the profunda femoris vein on preoperative CT. Despite negative Doppler ultrasound, surgical exploration revealed a 3 mm venous laceration, which was repaired. The fragment was resected before fracture fixation with an intramedullary nail. Postoperatively, the patient developed deep vein thrombosis, managed with an inferior vena cava filter and anticoagulation.

**Conclusions:**

This case illustrates that a displaced lesser trochanteric fragment in proximity to major vessels may carry a risk of intraoperative venous injury, even in the absence of preoperative signs. It highlights the importance of preoperative imaging review for anatomical risk assessment. While this single case cannot establish management protocols, it contributes to the awareness of venous injury as a potential complication in similar fracture patterns.

## 1 Introduction

Vascular injury associated with intertrochanteric fractures is uncommon, with an incidence of approximately 0.2%, and predominantly involves arterial structures such as the profunda femoris artery (PFA) or its branches (1–3). Injuries specifically attributable to a displaced lesser trochanteric fragment are even rarer, with most reported cases describing arterial pseudoaneurysms or lacerations (4–6). In contrast, venous injuries—particularly involving the profunda femoris vein (PFV)—are scarcely documented, and cases in which a sharp bony fragment lies in direct contact with a major vein without preoperative evidence of injury are exceptionally uncommon (7–9). This case report presents a detailed account of an intraoperative PFV laceration caused by a displaced lesser trochanteric spike, with the aim of contributing to the limited literature on venous vascular risk in hip fracture surgery.

## 2 Case presentation

### 2.1 Patient information

An 83-year-old male patient was admitted to our hospital with severe left hip pain and functional impairment following a fall at home. On clinical examination, the patient was unable to bear weight on the left leg, which appeared shortened and externally rotated. Manipulation of the limb worsened groin pain. There was no neurovascular deficit. Medical history included a previous cerebral infarction, with ongoing left-sided limb weakness. The patient was on a daily regimen of aspirin 100 mg and walked slowly with the aid of crutches. No other major systemic comorbidities (such as diabetes, hypertension, or coronary artery disease) were present. After the injury, the patient and his family, fully aware of the risk of fracture, initially declined the recommendation for hospitalization and surgery, hoping to observe the situation at home first. However, during the period at home, the pain persisted without relief, and functional limitations became severe. Recognizing that conservative management was ineffective, the patient and his family proactively returned to the hospital on the 11th day post-injury to seek further treatment.

### 2.2 Diagnostic assessment

#### 2.2.1 Diagnostic methods

**Figure 1:**
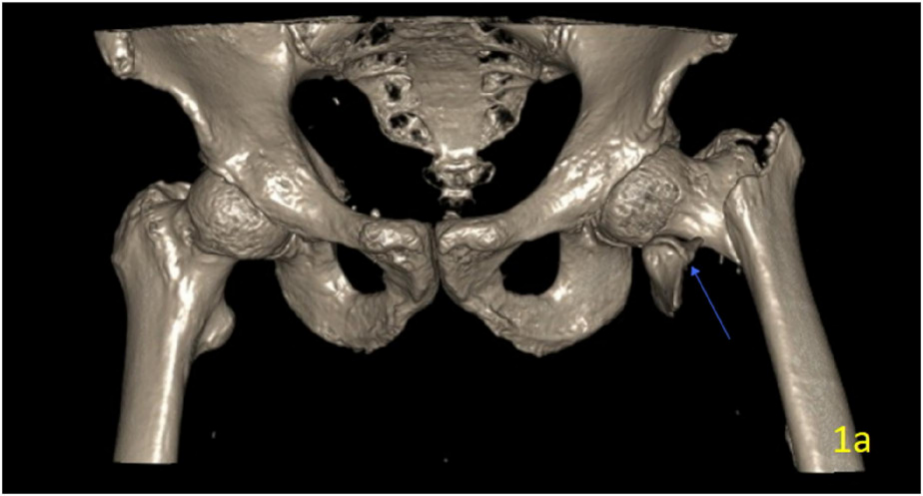
Preoperative 3D-CT reconstruction of the left hip. The image shows a markedly displaced intertrochanteric femoral fracture, classified as 31-A2.2 according to the Orthopaedic Trauma Association (AO/OTA 31-A2.2).

**Figure 2:**
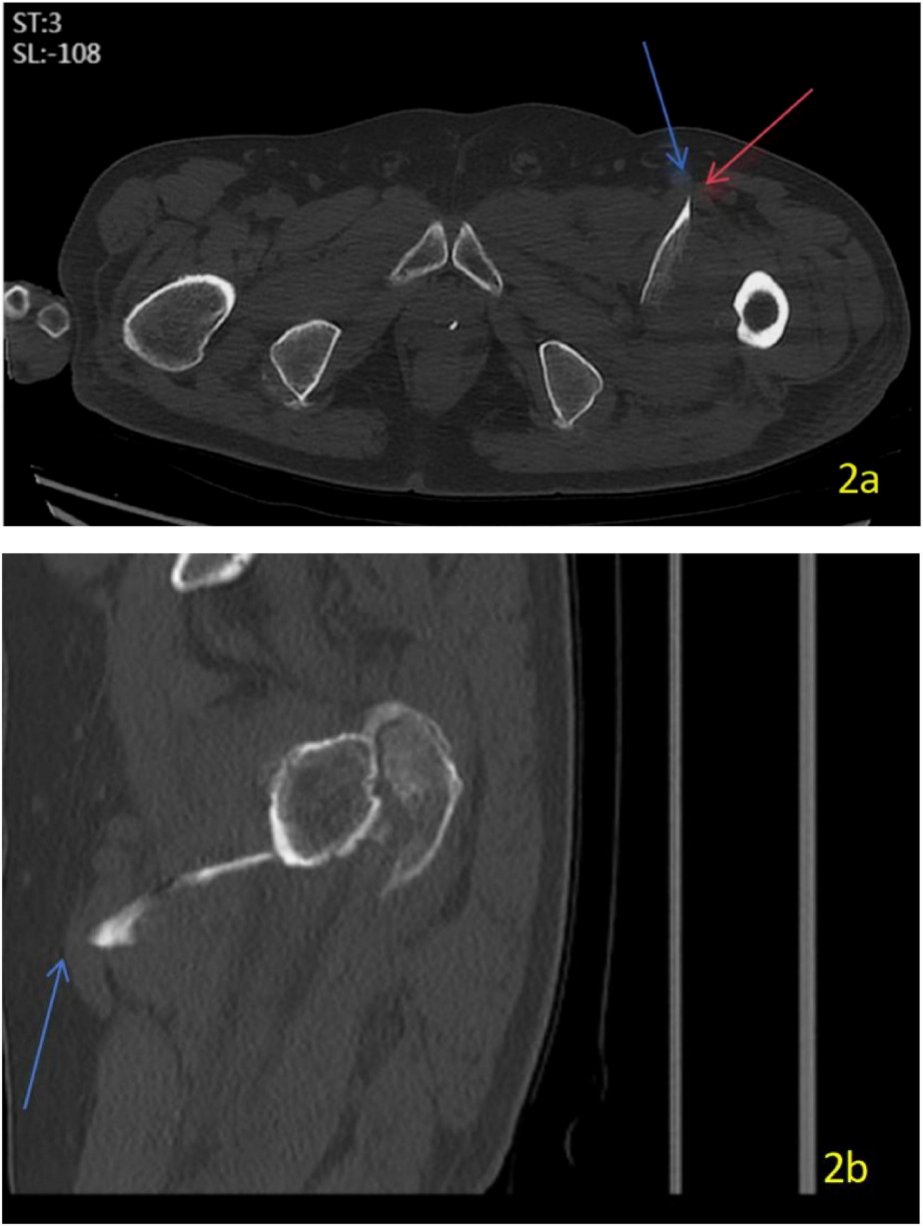
Two-panel CT series of the left hip. The left image (**a**, horizontal view) shows an anteriorly displaced bone spike from the lesser trochanter adjacent to vascular structures, indicated by a red arrow and a blue arrow. The right image (**b**, sagittal view) also shows this forward displaced spike adjacent to a vessel, marked by a blue arrow. This close relationship was the key imaging finding that prompted concern for potential vascular compromise.

After admission, complete the relevant examinations. Computed tomography (CT) of the affected hip was performed using a multidetector scanner with the following parameters: slice thickness 1.0 mm, reconstruction interval 0.8 mm, tube voltage 120 kV, and automatic tube current modulation. The scan revealed an intertrochanteric fracture with a sharp fragment of the lesser trochanter displaced forward and inward. The fracture was classified as AO/OTA 31-A2.2, indicating an intertrochanteric fracture with a single intermediate fragment and involvement of the lesser trochanter as a separate, displaced fragment (Figure 1). 3D CT scan revealed a displaced bony spike from the lesser trochanter, directed anteriorly towards the PFV and lateral to the PFA (Figures 2a,b). Preoperative color Doppler ultrasound of the left lower limb was performed, which showed no evidence of vascular laceration or pseudoaneurysm. On admission, laboratory tests revealed a hemoglobin level of 128 g/L, with all other values within the normal range for the patient's age. Serial hemoglobin monitoring showed a trend: 118 g/L on day 2 and 115 g/L on day 3 of admission.

#### 2.2.2 Diagnostic challenges

The primary diagnostic challenge centered on the discrepancy between imaging findings and functional vascular assessment. While 3D-CT clearly delineated a sharp, anteromedially displaced lesser trochanteric spike in intimate anatomical relationship with the PFV, the routine color Doppler ultrasound failed to detect any vascular injury. This created a clinical dilemma: the presence of a high-risk anatomical configuration without corroborative evidence of active vascular compromise. Furthermore, given the patient's advanced age, history of cerebral infarction and the desire to avoid the risk of contrast-induced nephropathy, computed tomography angiography (CTA)—which might have provided additional vascular detail—was deferred after a shared decision-making process with the patient and his family. Consequently, the diagnostic assessment relied on interpreting the significance of a potentially hazardous bony fragment in the absence of definitive pre-operative vascular imaging.

#### 2.2.3 Clinical reasoning and preoperative planning

Based on the diagnostic findings and challenges, our clinical reasoning proceeded as follows: the intimate contact between the sharp bony spike and the PFV, as unequivocally demonstrated on CT, posed a tangible and unacceptable risk of venous laceration—either pre-existing, induced during patient movement, or occurring intraoperatively during fracture manipulation or fixation. A negative Doppler ultrasound was not considered sufficient to exclude this mechanical risk, as it primarily assesses luminal flow and may not detect wall contusion or adherence. Therefore, despite the absence of preoperative signs of vascular injury, the high-risk anatomy warranted proactive management. Our surgical plan was thus designed to address the vascular risk definitively before fracture fixation. We prioritized direct intraoperative exploration as the conclusive diagnostic and therapeutic step for any vascular compromise. The decision logic was to first secure vascular safety (explore, repair if needed, and resect the offending fragment) in a controlled manner, thereby eliminating the source of potential catastrophic hemorrhage before proceeding with the biomechanically demanding steps of fracture reduction and nailing.

### 2.3 Surgical intervention and intraoperative findings

**Figure 3:**
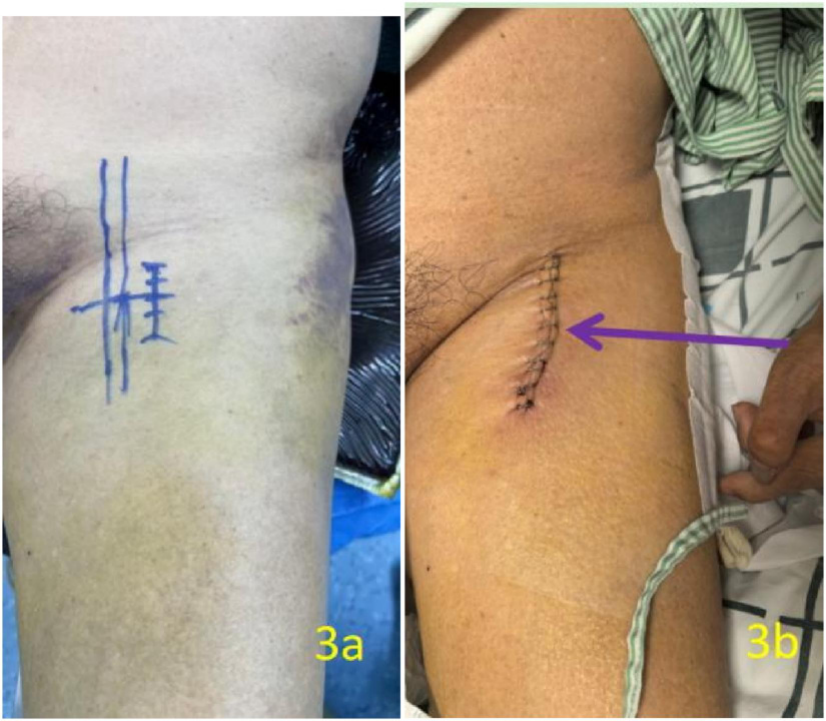
Two photographs of the left inguinal region. The left image (**a**, preoperative) shows planned surgical incision lines drawn on the skin. The right image (**b**, postoperative) shows the resulting 8 cm longitudinal incision.

**Figure 4:**
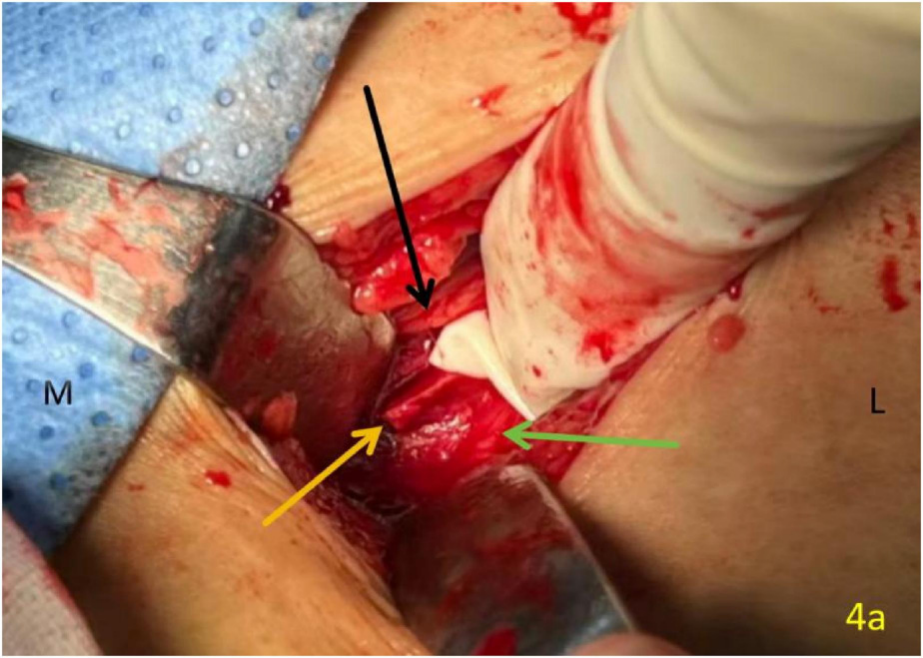
Intraoperative photograph of the left inguinal region. A displaced bone spike (orange arrow) is exposed and seen in direct contact with the wall of an artery (green arrow) and a vein (black arrow). The main femoral artery appears intact.

**Figure 5:**
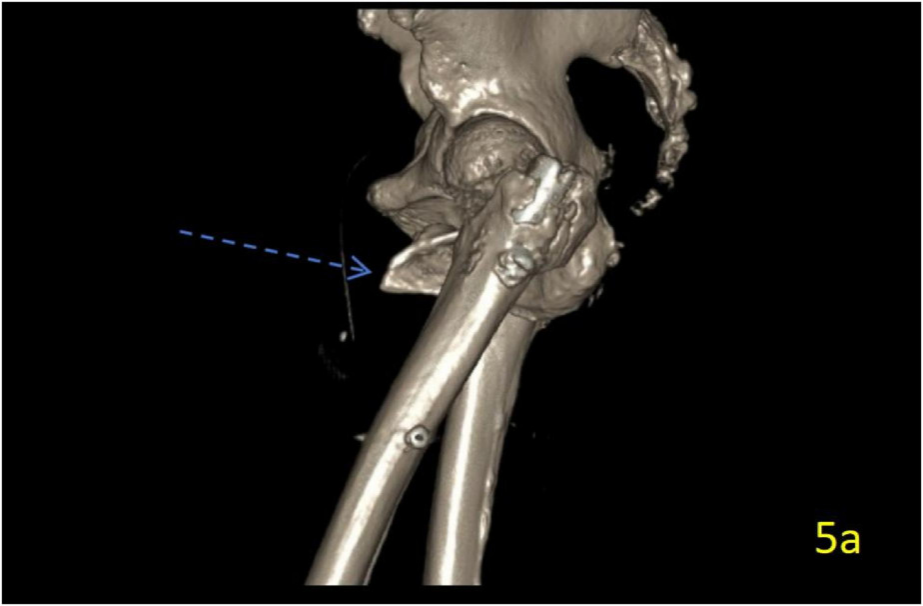
Lateral view of a postoperative 3D-CT scan of the left hip. Comparison with preoperative imaging confirms the resection of the previously displaced lesser trochanteric spike.

From admission to preoperative period, the patient received prophylactic low molecular weight heparin (LMWH, 4,000 IU once daily) to balance thromboprophylaxis with the increased bleeding risk associated with stroke history and ongoing antiplatelet therapy. Two days after admission, the patient was positioned supine on a traction bed under general anaesthesia with tracheal intubation, ensuring that no excessive traction was applied. No attempt was made to mobilise the lesser trochanter fragment or introduce instruments anterior to the hip joint that might cause iatrogenic vascular injury. The surgical approach was planned based on preoperative CT imaging, which showed the displaced fragment located in the left inguinal region medial to the femoral artery. After routine disinfection and sterile draping, an 8 cm longitudinal incision (Figures 3a,b) was made in the left inguinal region. Upon incising the skin and subcutaneous tissue, the surgeon's finger was introduced to palpate and localize the sharp bony fragment. This finger was positioned anterior to the fragment to act as a protective shield between the bone spike and the underlying femoral vessels throughout the subsequent dissection. The displaced spike of the lesser trochanter was exposed and found to be in direct contact with the wall of the PFV (Figure 4). The PFA was continuous and intact. However, careful inspection revealed a pre-existing linear tear of approximately 3 mm in the venous wall at the site of adhesion to the bony spike. During the subsequent meticulous blunt dissection to separate the fragment from the vein, this pre-existing tear was encountered and slightly extended. The defect was repaired directly using 5-0 Prolene sutures. The PFA was continuous and intact throughout the procedure. The spike of the lesser trochanter, located adjacent to the PFV and PFA, was resected (Figure 5). The wound was closed without placement of a suction drain. The intertrochanteric fracture was then managed with closed reduction and internal fixation using an InterTAN intramedullary nail (Model: InterTAN; Manufacturer: Double Medical Technology, Inc., Xiamen, China) on a traction table. The patient received a transfusion of two units of blood during the operation.

### 2.4 Postoperative course and follow-up

**Figure 6:**
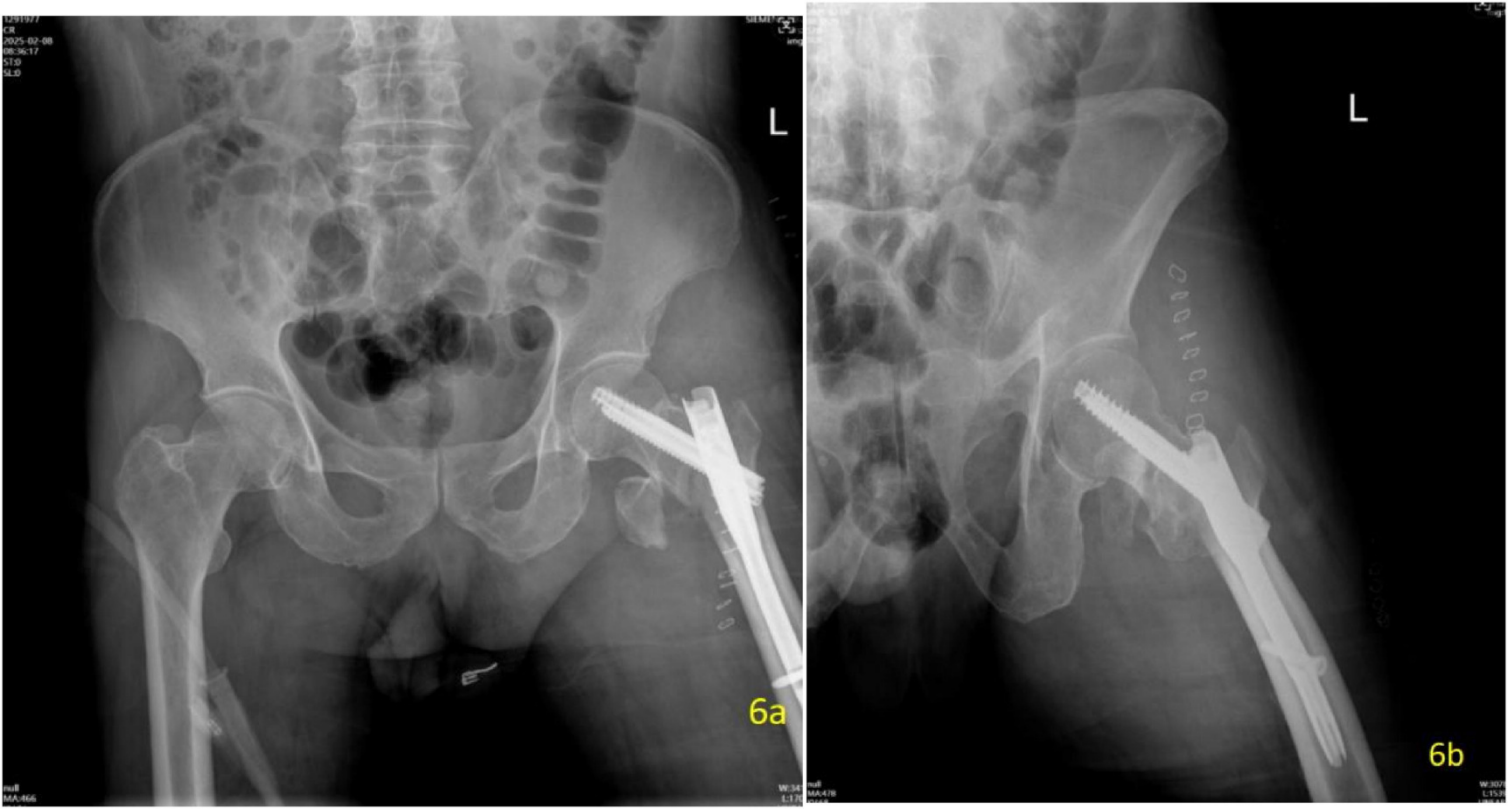
Two-panel postoperative x-ray series of the left hip on day 1. The left image (**a**, anteroposterior view) and the right image (**b**, lateral view) confirm satisfactory fracture reduction and positioning of the internal fixation implant.

Postoperatively, vital signs remained stable. Postoperative x-ray indicated good reduction of fractures (Figures 6a,b). Mild pain and swelling were present in the left hip, and the patient was able to move the limb actively. Postoperative hemoglobin levels were monitored closely: 100 g/L at 2 h post-operation, 93 g/L on postoperative day 1, and 85 g/L on postoperative day 4. Re-examination confirmed that the hemoglobin level remained stable which indicated without bleeding of rupture of the PFV. After the operation, the patient received a preventive dose of low-molecular-weight heparin (LMWH, 4,000 IU once daily) treatment. On postoperative day 4, the patient developed mild unilateral calf swelling. Colour Doppler ultrasound revealed thrombosis in the proximal superficial femoral vein (SFA). Therefore, the anticoagulant dose was increased to the therapeutic dose LMWH (4,000 IU twice daily). Given the presence of proximal SFA thrombosis and the associated high embolic risk, an inferior vena cava (IVC) filter was placed on postoperative day 7. Following the procedure, the patient was treated with subcutaneous low-molecular-weight heparin (4,000 units twice daily) during hospitalization until discharge on February 17. After discharge, anticoagulation was switched to rivaroxaban 15 mg orally twice daily. This regimen was continued until follow-up ultrasound on March 20 confirmed resolution of the left SFA thrombosis. The patient was readmitted on March 20, and the IVC filter was successfully removed on March 21. No evidence of pulmonary embolism was identified on preoperative or postoperative imaging studies. Hemoglobin levels subsequently improved: 93 g/L on postoperative day 6, rising to 140 g/L by postoperative day 13, and thereafter remained stable.

**Figure 7:**
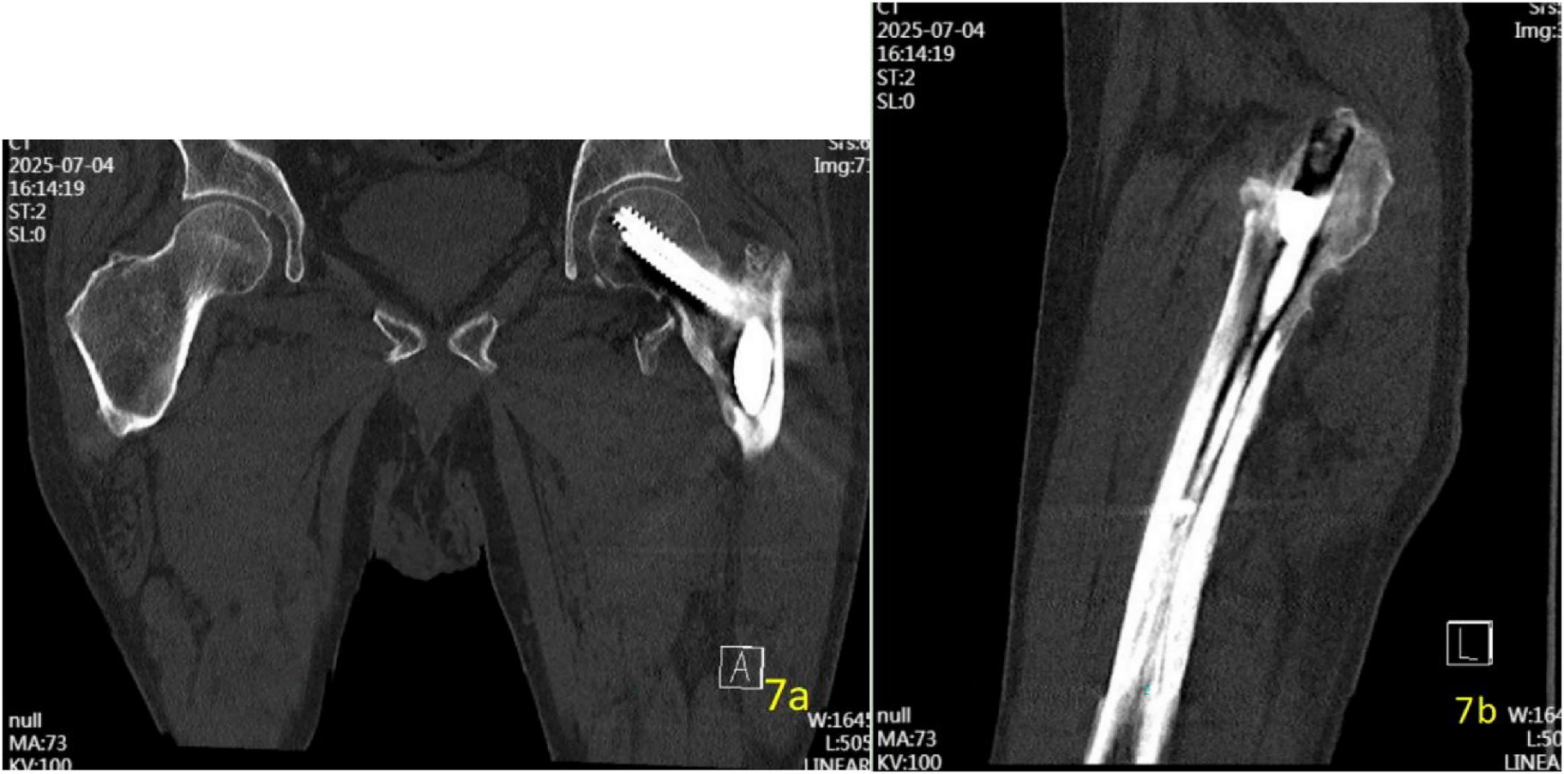
Two-panel CT scan series at the 5-month follow-up. The left image (**a**, coronal reconstruction) and the right image (**b**, sagittal reconstruction) demonstrate complete bony union of the intertrochanteric fracture.

The patient remained clinically well and reported no significant complications. One week after the second operation, the patient was discharged and continued rehabilitation at home. Clinical recovery was rapid, and hemoglobin values returned to normal after 4 weeks. One month later, the IVC filter was removed successfully. The patient was followed up regularly for 5 months. At the last follow-up, the patient reported minimal pain (VAS 1/10 at rest), walked with a single cane for household distances (>100 m), and had no residual thigh swelling or neurological symptoms. Radiographic union was complete (Figures 7a,b). The patient expressed overall satisfaction with the functional outcome despite the complex recovery involving two procedures, and was particularly appreciative of the transparent communication and comprehensive care provided throughout the treatment pathway. Table 1 shows the Timeline of Clinical Events.

**Table 1:** Timeline of clinical events.

Date/Time point	Event description	Notes
Injury day (day 0)	Patient fell at home, resulting in severe left hip pain and inability to bear weight.	Clinical examination showed left leg shortening, external rotation, and groin pain.
Day 11 post-injury	Patient and family proactively returned to hospital due to ineffective conservative management (persistent pain, severe functional limitation).	Had initially declined hospitalization and surgery.
On admission	Completed examinations: CT revealed left intertrochanteric femoral fracture (AO/OTA 31-A2.2) with an anteromedially displaced lesser trochanteric spike adjacent to profunda femoris vessels; Color Doppler ultrasound showed no vascular laceration or pseudoaneurysm. Lab tests showed hemoglobin 128 g/L.	CTA was deferred after shared decision-making due to patient's advanced age, history of cerebral infarction, and risk of contrast-induced nephropathy.
Admission to pre-op	Discontinued aspirin, switched to prophylactic dose subcutaneous LMWH (4,000 IU once daily).	A “bridging anticoagulation” strategy to balance stroke prevention and surgical bleeding risk.
Post-admission day 2	Surgery Day: Under general anesthesia, an 8 cm longitudinal incision was made in the left inguinal region for exploration. The displaced lesser trochanteric spike was found in direct contact with the PFV wall, causing a ∼3 mm laceration.	Intraoperative steps: (1) Direct repair of venous laceration with 5-0 Prolene suture; (2) Resection of the spike; (3) Wound closure followed by closed reduction and internal fixation with an InterTAN intramedullary nail. Received 2 units of blood transfusion.
Immediately post-op	Vital signs stable. Repeat hemoglobin stable. x-ray confirmed satisfactory fracture reduction.	Continued prophylactic LMWH (4,000 IU once daily).
Post-op day 4	Developed mild unilateral calf swelling. Color Doppler ultrasound confirmed proximal superficial femoral vein (SFA) thrombosis.	Anticoagulation escalated to therapeutic dose LMWH (4,000 IU twice daily).
Post-op day 7	Inferior vena cava (IVC) filter placement to prevent pulmonary embolism.	Decision based on high embolic risk from proximal DVT, patient age, and surgical trauma.

## 3 Discussion

Femoral vessel injuries associated with hip fractures are uncommon (10–13). In most reported cases, the vascular injury is iatrogenic, typically involving the SFA and caused by a protruding screw from an intra- or extramedullary implant used in the fixation of intertrochanteric fractures (1, 2, 11, 14–16). Less frequently, injury is caused by a displaced fragment of the lesser trochanter (3, 7–9). The primary diagnostic challenge lies in the preoperative recognition of such risk. Although 3D-CT in our case clearly delineated the fragment's proximity to the PFV, routine Doppler ultrasound was negative. This underscores a critical point: in complex fractures with significant lesser trochanter displacement, normal vascular studies do not preclude the risk of injury from direct bony contact. This case reinforces that a high index of suspicion based on CT findings is warranted, even in the absence of preoperative vascular signs.

It is pertinent to distinguish between arterial and venous injuries in this context. Table 2 summarizes key features of recently reported cases involving vascular injury from a displaced lesser trochanter, highlighting the predominance of arterial involvement. Arterial injuries, such as laceration or pseudoaneurysm of the PFA or its branches, often present more dramatically with expanding hematoma, pulsatile mass, or distal ischemia, and may require urgent intervention to control hemorrhage or restore perfusion (7, 9, 16–20). Venous injuries, like the PFV laceration encountered in our case, are reported less frequently and may initially be subtle, presenting primarily with occult bleeding or later as deep vein thrombosis. A displaced lesser trochanter has also been reported in relation to iatrogenic damage to the SFA in the literature (3, 6, 21–23). The intraoperative discovery of a PFV laceration caused by direct bony contact, as in our patient, is particularly rare in the literature. Our case adds to this sparse literature by documenting a venous injury that was asymptomatic preoperatively but posed a significant intraoperative risk. The finding of a pre-existing venous tear upon exploration confirms the fragment itself as the injury mechanism, underscoring that normal preoperative assessment does not eliminate the risk posed by an adherent bony spike.

**Table 2:** Reported cases of vascular injury associated with a displaced lesser trochanter fragment.

Author(s), year	Injured vessel	Injury mechanism	Treatment	Key findings
Potenza et al., 2016 (7)	Branch of PFA	Laceration by bone spike	Ligation	Pre-op CT showed proximity; injury found during open fragment removal.
Mayurasakorn et al., 2017 (8)	PFA	Pseudoaneurysm	Endovascular coiling	Presented with delayed thigh swelling and anemia.
Čičak et al., 2025 (9)	Deep femoral artery	Delayed injury from migrated fragment	Endovascular stent-graft	Highlighted risk of fragment migration post-fixation.
Osagie et al., 2015 (3)	Profunda femoris artery	Injury during IM nailing	Surgical repair	Emphasized iatrogenic risk from surgical maneuver.
Current case	Profunda femoris vein (PFV)	Direct laceration by adjacent spike	Direct suture repair	Rare venous injury; pre-op imaging showed contact without leak; managed prophylactically.

This patient presented a specific perioperative anticoagulation challenge due to his history of cerebral infarction and prior aspirin use. To balance thrombotic risk against surgical bleeding, we employed a bridging strategy: aspirin was discontinued, and prophylactic low-molecular-weight heparin (LMWH) was initiated preoperatively. This approach aimed to maintain antithrombotic protection while mitigating the prolonged antiplatelet effect of aspirin during surgery. Postoperatively, the patient developed a proximal superficial femoral vein thrombosis despite prophylactic LMWH, necessitating escalation to therapeutic anticoagulation and placement of an inferior vena cava filter. This clinical course underscores the multifactorial and heightened hypercoagulable state commonly encountered in elderly patients following hip fracture surgery, which is attributed to factors such as surgical trauma, immobility, pre-existing vascular disease, and the underlying fracture itself. While the repaired venous injury at the PFV site may have contributed to local stasis or endothelial inflammation, the development of thrombosis in a separate venous segment (the SFA) suggests that systemic and anatomical risk factors likely played a predominant role. This observation highlights that postoperative DVT in such complex cases is rarely attributable to a single cause but rather emerges from a confluence of patient-specific and procedural factors. It also underscores the need for close monitoring and potentially individualized thromboprophylaxis strategies in high-risk patients.

The management of the displaced lesser trochanter fragment itself warrants discussion. In our case, preoperative CT unequivocally demonstrated the sharp spike in dangerous proximity to the PFV. We therefore chose a proactive, open anterior approach to directly visualize, control, and resect the fragment before proceeding to fracture fixation. This decision was driven by the need to eliminate the source of potential catastrophic hemorrhage in a controlled setting. Alternative strategies, such as percutaneous manipulation or leaving the fragment unresected, were considered but deemed unsafe given the direct vascular abutment. The open approach allowed for primary venous repair when the tear was encountered and ensured complete removal of the offending bone. This initial vascular exploration and repair directly influenced the subsequent orthopedic strategy: we adopt the closed reduction technique and choose the InterTAN intramedullary nail precisely because its standard large rotor tip insertion point can completely avoid the vascular surgical area in front, achieving a safe separation of “vascular treatment” and “fracture fixation” in the operating space.

The timing of intervention is another important consideration. In the present case, the 10-day delay from injury to surgery may have increased the risk of further fragment migration or venous wall adherence, potentially complicating surgical dissection. Indeed, migration of the lesser trochanter fragment—facilitated by iliopsoas traction during patient movement or rehabilitation—is a recognized mechanism of vascular injury. Such injuries, if they occur post-operatively, carry not only clinical morbidity but also medicolegal risk. Previous literature supports early active intervention when a displaced lesser trochanter is identified in proximity to major vessels, even in the absence of overt vascular signs, to preempt such complications. Our experience reinforces that a proactive, open surgical approach not only allows direct treatment of any vascular injury but also definitively addresses the underlying bony cause through fragment resection, thereby mitigating both immediate and delayed risks.

Several key implications arise from this experience. First, meticulous review of preoperative CT scans for vascular proximity is essential in intertrochanteric fractures, especially those with significant lesser trochanter displacement. Second, when high-risk anatomy is identified, surgeons should have a low threshold for considering formal vascular imaging consultation or planning for intraoperative vascular exploration. Third, a multidisciplinary approach involving orthopedic and vascular expertise, either in preparation or on standby, is advisable for such complex cases. Finally, clear communication with the patient and family about the specific risks of vascular injury and the rationale for a potentially more extensive procedure is crucial for informed consent, particularly in elderly comorbid patients.

The current study has several limitations. Firstly, the omission of a preoperative radiograph, though compensated by detailed CT, may have initially underappreciated the risk posed by the anteromedially displaced fragment. In standard practice, radiographs serve as an initial screening tool; their absence could delay recognition of high-risk fracture morphology in settings where advanced imaging is not immediately available. Secondly, while 3D-CT provided excellent bony detail, a formal CTA was not performed. Although this decision was made prudently based on patient-specific comorbidities and shared decision-making, it represents a limitation in the preoperative vascular assessment. A CTA might have provided additional information on the vessel wall contour and exact spatial relationship, potentially further stratifying the risk. Therefore, our recommendation for proactive exploration is based primarily on intraoperative findings and retrospective bony anatomy analysis, rather than prospective vascular imaging correlation. Finally, as a single case report, our findings, while instructive, cannot establish incidence or definitive treatment protocols. The conclusions require validation in larger series.

## 4 Conclusion

In this case, a displaced lesser trochanteric fragment adjacent to the profunda femoris vein was associated with an intraoperative venous laceration despite unremarkable preoperative vascular imaging. This finding suggests that close anatomical proximity between a sharp bone fragment and major vessels, as visualized on CT, may indicate a risk of vascular injury during surgery. While our experience is limited to a single case and does not support generalized recommendations, it underscores the value of meticulous preoperative planning and consideration of vascular risk in similar high-anatomy-risk configurations. Further observational studies are needed to better define the incidence and optimal management of such injuries.

## Data Availability

The original contributions presented in the study are included in the article/Supplementary Material, further inquiries can be directed to the corresponding author.
